# Diabetic Corneal Neuropathy: Pathogenic Mechanisms and Therapeutic Strategies

**DOI:** 10.3389/fphar.2022.816062

**Published:** 2022-02-23

**Authors:** Ting Zhou, Allie Lee, Amy Cheuk Yin Lo, Jeremy Sze Wai John Kwok

**Affiliations:** Department of Ophthalmology, Li Ka Shing Faculty of Medicine, The University of Hong Kong, Pokfulam, Hong Kong SAR, China

**Keywords:** corneal nerve damage, corneal confocal microscopy, diabetic complications, dry eye, nerve regeneration, ocular disease, ocular surface

## Abstract

Diabetes mellitus (DM) is a major global public health problem that can cause complications such as diabetic retinopathy, diabetic neuropathy, and diabetic nephropathy. Besides the reporting of reduction in corneal nerve density and decrease in corneal sensitivity in diabetic patients, there may be a subsequent result in delayed corneal wound healing and increased corneal infections. Despite being a potential cause of blindness, these corneal nerve changes have not gained enough attention. It has been proposed that corneal nerve changes may be an indicator for diabetic neuropathy, which can provide a window for early diagnosis and treatment. In this review, the authors aimed to give an overview of the relationship between corneal nerves and diabetic neuropathy as well as the underlying pathophysiological mechanisms of corneal nerve fiber changes caused by DM for improved prediction and prevention of diabetic neuropathy. In addition, the authors summarized current and novel therapeutic methods for delayed corneal wound healing, nerve protection and regeneration in the diabetic cornea.

## Introduction

Diabetes mellitus (DM) is a chronic metabolic disorder characterized by abnormally increased blood glucose level, which over time leads to severe systemic damage such as diabetic foot ulcers, nephropathy and neuropathy. According to World Health Organization (WHO), about 422 million people worldwide suffer from DM, and 1.6 million deaths are directly attributed to DM each year (WHO, 2021). Over the past few decades, the number of cases and prevalence of DM have been increasing steadily. Thus, the huge public health challenge and economic burden that many countries are about to face emphasize the importance of effective treatment and prevention for various complications associated with DM.

The complications of DM include cardiovascular disease, nephropathy, retinopathy and neuropathy (Markoulli et al., 2018). Among them, diabetic retinopathy has been well-recognized. Besides, it was reported that more than half of the patients were presented with alternation of anterior segment (such as cornea, conjunctiva, lacrimal gland and lens) during DM progression (Vieira-Potter et al., 2016; Barsegian et al., 2018); but these symptoms are sometimes overlooked. Indeed, DM patients have shown a progressive loss in corneal nerve fiber and a decrease in corneal sensitivity (Dogru et al., 2001; Pritchard et al., 2015), which subsequently led to delayed nerve regeneration and wound healing after injury, dry eye, persistent epithelial defects, and neurotrophic ulcers (Kaiserman et al., 2005; Pritchard et al., 2011). These complications may cause severe vision loss or even blindness, reminding us that it is vital to understand the effects of DM on corneal nerves (Bikbova et al., 2018; Han et al., 2018).

In this review, the authors outlined the association between DM and corneal nerves and summarized the current knowledge regarding the underlying pathophysiologic mechanisms of corneal nerve fiber changes caused by DM. Also, the authors explored current and the most recent therapeutic approaches for nerve protection and regeneration in the diabetic cornea.

## Diabetic Corneal Neuropathy

### Clinical Perspectives of Corneal Neuropathy in Diabetes Mellitus

The cornea is one of the most important parts of optical system. Its transparent and avascular physiological characteristics ensure clear vision (Al-Aqaba et al., 2019). The cornea is innervated by the trigeminal nerve; it indeed is the most densely innervated structure in the entire human body. The cornea is also one of the most sensitive body parts to pain as the number of free epithelial nerve endings are 300–600 times that of the skin. Cornea contains a large number of small nerve fibers like myelinated A-δ fibers and unmyelinated C fibers. The physiological function of A-δ fibers is cold sensation and nociception, while C fibers are mainly responsible for warm, cold, thermal perception (Körei et al., 2016). These sensory nerves also control reflex function and tear secretion. In addition, the corneal nerves play a crucial neurotrophic role through releasing neurotrophic factors to maintain integral and healthy structure and function of the ocular surface, and are the major determinant in maintaining ocular surface homeostasis, corneal sensitivity, epithelial health, and wound healing (Byun et al., 2015; Labetoulle et al., 2019).

It was reported that neuropathy of the cornea occurred in the early stage of DM (Zhao et al., 2019). The clinical symptoms in diabetic patients with corneal neuropathy are photophobia, eye irritation or pain (Bikbova et al., 2018). Some patients have no symptoms, but subclinical changes in the cornea may exist (Rehany et al., 2000; Scheler et al., 2012). Chronic hyperglycemia in diabetic patients can cause damage to the trigeminal nerve, leading to a reduction or loss of corneal nerves, most of which are damages to A-δ and C nerve fibers. Damage or loss of corneal innervation can in turn lead to reduced corneal nerve fiber density, attenuated corneal sensitivity, dry eyes, delayed wound healing, subsequently developing into corneal ulcers, perforations, and even blindness (Shaheen et al., 2014).

*In vivo* corneal confocal microscopy (CCM) is an essential method to investigate the fine structure of corneal nerves in detail. It provides real-time visualization of corneal nerve fibers in a non-invasive way, allowing long-term longitudinal studies in both patients and animal models (Leckelt et al., 2016). The acquired corneal images can be analyzed by appropriate software for the identification of quantitative indicators (Schaldemose et al., 2020). Using CCM, several published studies reported corneal nerve fiber changes in diabetic patients. Whether it is type 1 diabetes (TID) or type 2 diabetes (T2D), the nerve fiber length, nerve fiber density and nerve branch density in these patients were significantly reduced (Chen et al., 2018; Li et al., 2019; Lewis et al., 2020). Lagali et al. revealed that as type 2 diabetes continued, the sub-basal nerve plexus showed a progressive degeneration (Lagali et al., 2017). They found a reduction of the main nerve branches, an apparent loss of the secondary nerve fiber branches as well as a loss of the connection with the primary nerve. In addition, one study examined the correlation between corneal nerve parameters and glucose variability. It explored whether short-term measures of blood glucose control were related to structural and functional alternations of corneal nerve in TID patients and found that significant loss of corneal nerve length in the inferior whorl occurred in patients with increased glucose variability and time above range (Issar et al., 2020). Moreover, the decrease in corneal keratocyte density was associated with damage in the sub-basal nerve plexus in diabetic patients (Kalteniece et al., 2018a). Other studies found that the density of the sub-basal nerve plexus in diabetic patients was attenuated while the density of Langerhans cells was increased (Qu et al., 2018; Ferdousi et al., 2019). These changes (lower corneal keratocyte density and higher Langerhans cells density) were related to delayed corneal epithelial healing (Qu et al., 2018). These data can help clinicians to better understand when and how diabetic corneal neuropathy develops and monitor cornea alternation and its progression.

Many clinical studies also explored the association between diabetic corneal nerve damage and complications of DM with CCM. Several studies demonstrated that changes in corneal nerve fibers were related to the development of diabetic retinopathy (DR). One study reported that in T2D patients, corneal nerve damage preceded the development of DR (Bitirgen et al., 2014). In TID patients with a four-year development of DM with/without DR, it was found that their corneal nerve fiber length was one of the predictors for worsening of DR (Srinivasan et al., 2018). The authors also reported that neuronal degeneration of both cornea and retina may occur in early stage of DR, so examination of corneal nerve was necessary especially when clinical signs of DR was absent (Srinivasan et al., 2017; Srinivasan et al., 2018). Meanwhile, reduction of corneal nerve fiber density, corneal nerve fiber length and nerve fractal dimension occurred in T2D patients with diabetic nephropathy. These corneal alternations were related to the decline of kidney function (Tummanapalli et al., 2020a). There were more clinical studies that focused on the damage of corneal nerve in diabetic neuropathy (DN). Edwards et al. demonstrated that the length and density of corneal nerve fibers in diabetic patients with mild DN were significantly reduced (compared to the control group and diabetic patients without DN) (Edwards et al., 2012). Similarly, other studies have reached consistent conclusions. They stated that the parameters of corneal nerve fibers could predict the development of DN and are a potential clinical biomarker for evaluating DN (Deák et al., 2019). Compared with controls, the corneal parameters of T1D and T2D patients, such as nerve fiber density, branch density, and nerve fiber length, were significantly reduced, and these indicators progressively worsened when the severity of DN increased (Kalteniece et al., 2020). The corneal nerve fibers in the inferior whorl region of diabetic patients were shown to be more sensitive to the damage than those in the central cornea, suggesting that the reduction of corneal nerve fibers is more significant in the inferior region (Kalteniece et al., 2018b; Ferdousi et al., 2020). Moreover, corneal nerve fiber damage was more severe in patients with painful neuropathy when compared with painless neuropathy (Kalteniece et al., 2018b). The results obtained from all these studies indicated an important association of corneal nerve with DM while corneal nerve fibers may be a reproducible biomarker for detection and assessment of early nerve damage in diabetes.

Of note, current diagnosis tests for DN patients without obvious symptoms include filament test, quantitative sensory test, nerve conduction test, muscle response test (electromyography), and autonomic test. All of these are tests designed for assessing large nerve fiber function (Selvarajah et al., 2019). However, impairment of small nerve fibers (type C and A-δ nerve fiber) is considered the earliest alteration in the course of DN, which progresses to large nerve fiber (Körei et al., 2016). Since the gold standard test for small fiber neuropathy is skin biopsy with intraepidermal nerve fiber density analysis, this invasive procedure makes it hard to implement in routine clinical practice (Basantsova et al., 2019). By the time when DN is detected with the aforementioned tests for large nerve fibers, it is very often well established already; but the neuropathic process can no longer be reversed. In conclusion, the detection of corneal nerve fiber with CCM may provide an open window for diagnosis of DN. It may potentially be a biomarker for the progression, or even treatment efficacy of DN. This emphasizes the importance of understanding the pathogenic mechanisms of corneal neuropathy in diabetes.

### Pathogenesis

**FIGURE 1:**
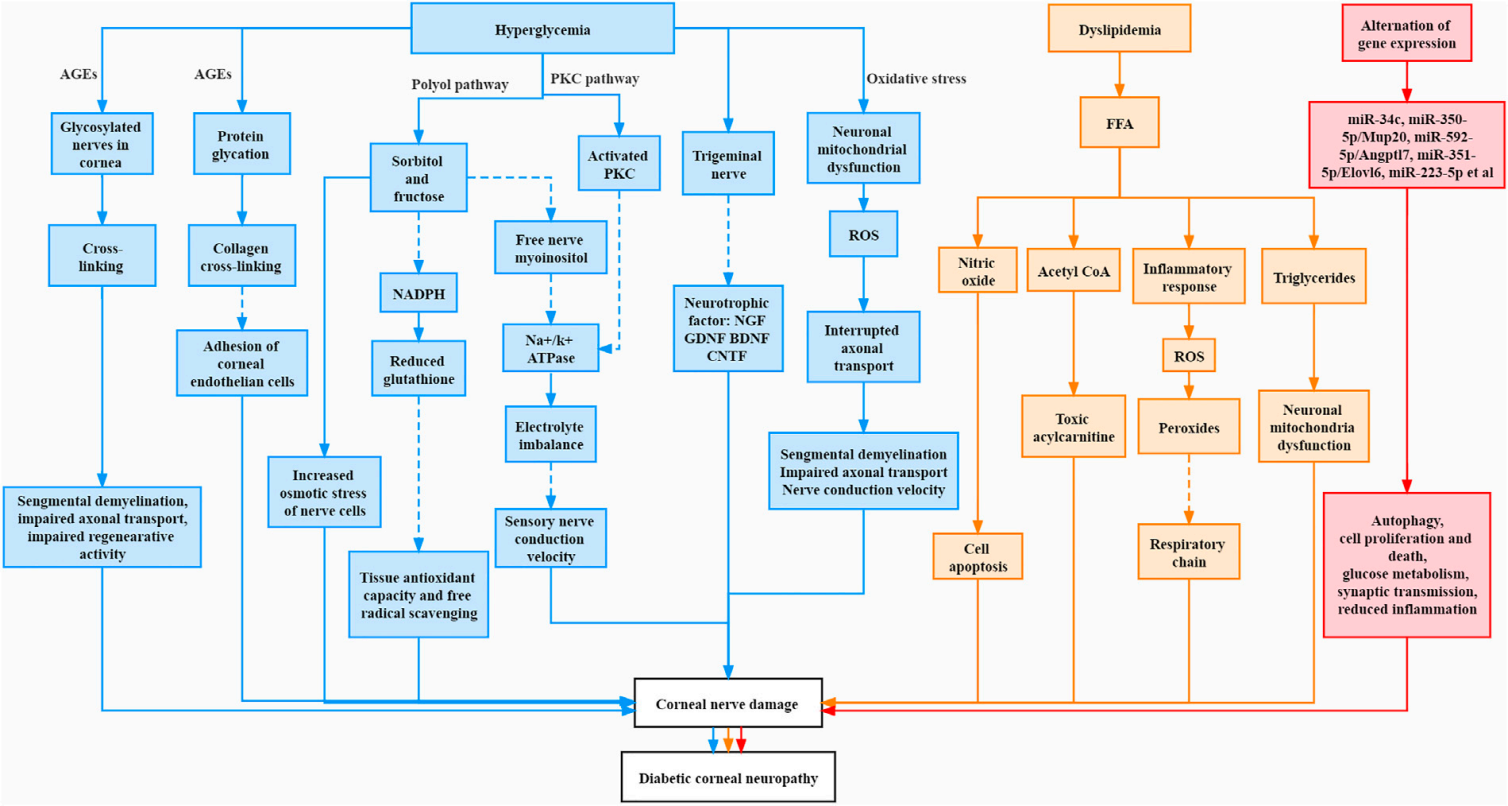
Pathogenesis of corneal neuropathy in diabetes mellitus. The Solid line represents increase and promotion, and the dashed line represents decrease and inhibition.

Many pathological changes were involved in diabetic corneal neuropathy, which interplayed and complemented each other, leading to abnormalities in cornea, including severe damage to corneal nerves (Roszkowska et al., 2021). The mechanism is summarized in Figure 1.

#### Hyperglycemia

##### Advanced Glycation End Products

Advanced glycation end products (AGEs) are reactive metabolites produced by the glycation of proteins or lipids that are exposed to sugar molecules and form non-enzymatic covalent bonds with each other (Babizhayev et al., 2015). The structural and functional proteins of peripheral nerves were reported to be glycosylated and they underwent cross-linking over time in diabetic patients and diabetic animal models. This resulted in an elevation of AGE level and in turn led to segmental demyelination, impaired axon transport and impaired regenerative activity (Sugimoto et al., 2008). In addition, AGEs may cause cross-linking of collagen fibris (Sady et al., 1995). Kaji et al. reported that glycosylated laminin and fibronectin reduced cell adhesion of corneal endothelial cells, thus leading to abnormalities of the diabetic cornea (Kaji et al., 2001). Moreover, AGEs were shown to change the structure and function of corneal epithelium, stroma and basement membrane (Bejarano and Taylor, 2019).

##### The Polyol Pathway

The polyol pathway is also known as the sorbitol-aldose reductase pathway. Chronic hyperglycemia leads to excessive glucose in nerve cells, resulting in saturation of the normal glycolytic pathway. Excess glucose is then shunted to the polyol pathway; it is first converted to sorbitol and then fructose by intracellular enzymes reductase and sorbitol dehydrogenase, respectively (Stavniichuk et al., 2012). These two sugar molecules (sorbitol and fructose) tend to accumulate within nerve cells because nerve cell membrane is impermeable to them, thus increasing the osmotic stress of nerve cells (Oates, 2008). At the same time, during the reduction process, aldose reductase consumes the cofactor nicotinamide adenine dinucleotide phosphate (NADPH) and leads to a decrease of reduced glutathione production, tissue antioxidant capacity and free radical scavenging, thereby causing tissue damage (Lyu et al., 2019). In addition, excessive amounts of sorbitol and fructose results in the decrease of free nerve myoinositol, which is sugar alcohol required for normal nerve conduction velocity (Greene et al., 1992). A decrease in its level can lead to a reduction in membrane sodium potassium adenosine triphosphatase (Na^+^/K^+^ ATPase) activity, resulting in electrolyte imbalance, reducing nerve conduction velocity, and destroying the structure of nerve fibers (Zhu et al., 2019).

##### Protein Kinase C Activation Pathway

Protein kinase C (PKC) is also an important pathogenic mechanism. In DM, increased intracellular glucose level plays a role in the synthesis of PKC activator diacylglycerol. The activation of PKC inhibits Na^+^/K^+^ ATPase activity, thereby affecting nerve conduction and nerve regeneration (Geraldes and King, 2010). A study found that the use of the PKC inhibitor ruboxistaurin mesylate improved microvascular blood flow and nerve conduction, thereby alleviating the symptoms of DN and improving the life quality of diabetic patients (Casellini et al., 2007).

##### Neurotrophic Factors

Results of several *in vitro* studies indicated that there is a mutual trophic support between the corneal nerve plexus and the corneal epithelium. The corneal nerve plexus secretes neuropeptides to maintain epithelial homeostasis and enhance wound healing. The corneal epithelium releases neurotrophic factors, such as nerve growth factor (NGF) and glial cell line-derived neurotrophic factor (GDNF), which can accelerate epithelial wound healing and stimulate neurite survival (Bikbova et al., 2016; Barsegian et al., 2018). Brain-derived neurotrophic factor (BDNF) is found in both corneal epithelial cells and corneal neurons (Byun et al., 2015). Therefore, the corneal nerve is closely related to the corneal epithelium. Neuropeptides released by sensory nerves could support epithelial cells and dendritic cells in the cornea to maintain its homeostasis. Chronic hyperglycemia may affect the neuropeptide secretion from the corneal nerves, thereby affecting the proliferation of cells in cornea (Markoulli et al., 2017; Zhang J. et al., 2020). In the denervated cornea of ​​rats, the rate of mitosis of corneal epithelial cells was changed (Ferrari et al., 2011). Gao et al. discovered that the number of dendritic cells, the main source of ciliary neurotrophic factor (CNTF), in the diabetic cornea was reduced. They demonstrated that the neural communication of dendritic cells was affected, resulting in impaired innervation and regeneration of sensory nerves (Gao et al., 2016). Zhou et al. found that in diabetic mice, a reduction of ciliary neurotrophic factor (CNTF) caused impaired corneal epithelial wounds, while CNTF supplementation could improve this pathology (Zhou et al., 2015). These articles demonstrated that diabetes could affect the corneal nerves through the secretion of neurotrophic factors, and at the same time affect the function of the corneal epithelium.

##### Oxidative Stress

Oxidative stress refers to the phenomenon that cells and tissues contain excessively high levels of reactive oxygen species (ROS), and the biological system has limited ability to reduce these active products to non-toxic substances (Pizzino et al., 2017). During hyperglycemia, there is an excessive influx of glucose into the mitochondria accelerating the oxidative metabolism of glucose in the mitochondria and the production of superoxide ions, thereby promoting the production of ROS (Babizhayev et al., 2015). Mitochondrial fission has been reported to occur in the neurites in response to hyperglycemia, generating oxidative stress and activating caspase 3. This was an indication of hyperglycemia-induced neurite damage (Vincent et al., 2010). In T2D patients, the length and density of corneal nerve fiber were decreased together with expanded beads in corneal nerve fiber. The bead is a novel parameter for a composite of accumulated mitochondria, glycogen particles and vesicles, which might be a predictor of slow sensory nerve conduction velocity in DM (Ishibashi et al., 2016). Mitochondrial abnormalities might be initiated by interrupted axonal transport, causing mitochondria to aggregate or cluster. As swollen mitochondria were difficult to be transported compared to smaller mitochondria, this eventually led to axonal demyelination and conduction dysfunction (Hamid et al., 2014). Mitochondrial damage is also related to the reduction of neurotrophic factors such as NGF and neurotrophin-3 (NT-3) (Zherebitskaya et al., 2009). One study found that increased 8-hydroxydeoxyguanosine (a marker of oxidative stress) was detected in the cornea of diabetic rats when compared with the control group (Kim et al., 2011a). This indicated a potential role of oxidative stress in the apoptosis of diabetic corneal cells.

##### Neuro-Immune Interactions

Neuro-immune interactions may also play a role in diabetic corneal neuropathy. In diabetic mouse models, high concentrations of Langerhans cells and dendritic cells, the main antigen-presenting cells, were found in the cornea and they aggregated around corneal nerve fibers (Leppin et al., 2014). When compared with the control group, the number of dendritic cells and Langerhans cells of diabetic patients elevated significantly, which negatively correlated with the length and density of corneal nerve fibers (Tavakoli et al., 2011). Another clinical study reported that early immune activation and corneal nerve degeneration occurred in adolescents with T1D (Ferdousi et al., 2019). In addition, it was reported that DNase I could promote corneal nerve regeneration and corneal sensitivity through decreasing the neutrophils extracellular traps caused by neutrophil aggregation (Zhang J. et al., 2020). However, this view is controversial (Ljubimov, 2017), and no concrete conclusion has been reached yet. It is not clear whether the infiltration of immune cells in the cornea precedes the induction of hyperglycemia.

#### Lipids

In addition to the effects of long-term hyperglycemia, dyslipidemia may also be a key factor in neuropathy, especially in T2D. Firstly, dyslipidemia can lead to an excessive increase in the level of free fatty acid (FFA), which can cause an elevation in nitric oxide, thereby leading to cell apoptosis (Stickings and Cunningham, 2002). Secondly, FFA enters the mitochondria and is catabolized by *ß*-oxidation to generate acetyl CoA. Excessive accumulation of acetyl CoA after dyslipidemia facilitates its conversion into toxic acylcarnitine substances, leading to nerve injury. Also, *ß*-oxidation activates inflammatory cells to release cytokines and induces the production of ROS, resulting in the production of peroxides. The oxidation of low-density lipoprotein inversely induces the generation of ROS, inhibits the electron transfer in the respiratory chain and aggravates nerve injury, which in turn forms a vicious circle (Roselló-Busquets et al., 2019; Rumora et al., 2019). Also, it is reported that decreased corneal nerve fiber length has an inverse correlation with low-density lipoprotein cholesterol (LDL-C) in T1D patients (Ferdousi et al., 2021). Moreover, when excessive FFA exhausts the oxidizing capacity of tissues, they are markedly deposited in non-fat tissues in the form of triglycerides, causing damage to these tissues. This phenomenon is called lipotoxicity. It is worth noting that the ability of neuronal mitochondria to metabolize and consume fatty acids is very limited (Schönfeld and Reiser, 2013). High levels of active oxygen can easily induce fatty acid peroxidation. If neuronal cells cannot clear up these peroxidized fatty acids in time, pathological changes will occur in the neurons, leading to neuropathy (Sultana et al., 2013).

#### Alternation of Gene Expression

Recently, several studies focused on the difference of gene expression between normal and diabetic trigeminal ganglion tissues. It was reported that in the trigeminal nerve tissue of T1D mice, abnormal expression of miR-34c could decrease sensory neuron growth of trigeminal nerve and the repair of corneal nerve endings by regulating the autophagy-related proteins Atg4B and LC3-II, thereby causing corneal neuropathy (Hu et al., 2019). Another study showed that some miRNA-mRNA pairs, such as miR-350-5p/Mup20, miR-592-5p/Angptl7 and miR-351-5p/Elovl6, are related to diabetic corneal neuropathy. And they reported that dysregulated genes could affect cell proliferation and death, glucose metabolism, and synaptic transmission (Zhang et al., 2020a). Recently, they further identified the miR-223-5p/*Hpgds* axis after screening by RNA sequencing. They reported that *Hpgds* is the direct target gene of miR-223-5p, and downregulation of miR-223-5p could promote corneal nerve regeneration by ameliorating inflammation (Zhang et al., 2021).

These studies provided new investigations on the pathogenesis of corneal neuropathy in DM and suggested that these alternations may be targets for gene therapy on diabetic corneal neuropathy.

## Treatment

Strict glycemic control is a vital prerequisite for the treatment in diabetic complications. So far, treatment of diabetic corneal diseases is still symptomatic. Here, we summarized and discussed current and innovative treatments for diabetes-related corneal pathologies. The advantages and limitations of various treatments are summarized in Table 1.

**TABLE 1:** Clinical and experimental therapeutic strategies for anterior segment disorders associated with diabetes.

Treatment strategy	Therapeutic agents/procedures	Clinical or experimental	Advantages	Limitations	References
Glycemic control	Insulin, Exenatide and pioglitazone	Clinical and experimental (rat, mouse)	Prevent nerve loss, Increase epithelial wound healing	Controversial effects in T2D, Increase IGF-1, exacerbate retinopathy	Smith et al. (1999); Chen et al. (2013); Yorek et al. (2014); Song et al. (2016); Khan et al. (2020); Ponirakis et al. (2020); Ponirakis et al. (2021b)
	Pancreatic islet cell transplantation	Clinical and experimental (rabbit)	Improve glycemic stability Reduce DN occurrence	Life-long immunosuppressive agents, Surgical complications, Not cost-effective	Wallner et al. (2016); Foster et al. (2018); Senior et al. (2018); Vantyghem et al. (2019)
Pharmacological intervention	Lipid-lowering drugs: statins	Clinical and experimental (rat)	Anti-inflammation, Reduce DN progression	Controversial effects	Bhalla et al. (2015); Miao Wang et al. (2021)
	Lipid-lowering drugs: fibrates	Clinical and experimental (rat, mouse)	Increase corneal nerve density, Improve corneal sensitivity	Side effects in T2D patients	Matlock et al. (2020)
	Supplements of fatty acids and its metabolites: menhaden oil, resolvin D1, DHA	Clinical and experimental (rabbit, rat, mouse)	Reverse corneal nerve loss, Promote neurite outgrowth, Resist oxidative stress	Very high dosage for clinical translation, Cotreatment with enalapril, *a*-lipoic acid, salsalate showed better effects	Lee et al. (2015); Serhan et al. (2015); Coppey et al. (2020); Lewis et al. (2021)
	ARI: Ranirestat, CT-112, ONO-2235	Clinical and experimental (rat)	Promote epithelial regeneration, Improve corneal sensitivity	Controversial effects in clinical trials	Fujishima and Tsubota (2002); Nakahara et al. (2005); Takamura et al. (2013)
	Trophic factor supplements: NGF	Clinical and experimental (rabbit, mouse)	Promote corneal nerve regeneration, Recover corneal sensitivity, Promote epithelial wound healing	Limited sources, High cost, Severe side effects	Bonini et al. (2000); Apfel (2002)
	Trophic factor supplements: SP	Experimental (rabbit, rat, mouse)	Promote epithelial wound healing	Only have effects when co-treatment with IGF-1	Abdelkader et al. (2011); Rocha-Neto et al. (2019); Tummanapalli et al. (2019)
	Trophic factor supplements: C-peptide	Experimental (rat, mouse)	Increase sub-basal nerve density	Short biological half-life, Unclear mechanism, Difficult clinical translation	Jolivalt et al. (2015)
	Trophic factor supplements: GLP-1	Experimental (mouse)	Increase neurite outgrowth	Few studies on cornea	Himeno et al. (2011)
	Trophic factor supplements: VIP	Experimental (mouse)	Promote nerve regeneration, Promote epithelial wound healing	Few studies	Zhang et al. (2020b)
	Trophic factor supplements: ARA290	Clinical	Increase corneal nerve density	Few studies	Brines et al. (2015)
	Antioxidants or anti-inflammatory drugs: Naltrexone	Experimental (rat, mouse)	Promote corneal epithelial regeneration	Difficult ocular delivery system	Sassani et al. (2016)
	Vitamin B12	Clinical	Reduce DN occurrence and progression	Few studies on cornea	Fogagnolo et al. (2020)
	Vitamin D	Clinical and experimental (rat, *in vitro*)	Related to DN and corneal nerve, Up-regulate NGF	Few studies on cornea	Lv et al. (2015); Almurdhi et al. (2017); Alam et al. (2021)
Bariatric surgery	Anti-inflammatory procedure	Clinical	Improve corneal nerve fiber length, corneal nerve fiber density and nerve branch density	Surgical complications, Not cost-effective	Iqbal et al. (2021); Adam et al. (2021)
Gene therapy	Targets: c-met, MMP-10 Method: adenovirus, adeno-associated virus	Experimental (human diabetic organ-cultured cornea)	Improve corneal wound healing rate	Adeno-associated virus has low transgene expression than adenovirus	Saghizadeh et al. (2014); Kramerov et al. (2016)
	Targets: miR-34c, miR-181a Method: Subconjunctival injection	Experimental (mouse, human diabetic organ-cultured cornea)	Promote corneal nerve regeneration	Multiple targets, Unpredictable side effects	Hu et al. (2019); Hu et al. (2020)
	Targets: HMGB1; *fl2*; miRNAs like miR-205 and miR-146a; drug delivery; CaMKKβ and PGC-1α Method: nanoparticles	Experimental (rabbit, rat, mouse)	Increase corneal epithelial regeneration	No clinical studies	Saleh et al. (2020); Hou et al. (2021); Jessi Wang et al. (2021); Lavker et al. (2021); Tsai et al. (2021)

### Glycemic Control

Since chronic hyperglycemia can cause nerve damage through a variety of pathological mechanisms, strict glycemic control is believed to be an essential means to treat diabetes and reduce the progression of its related complications (Ponirakis et al., 2021a). Many drugs have been used to control blood glucose; they include oral drugs such as metformin, glucagon-like peptide-1 (GLP-1) receptor agonist or sodium–glucose cotransporter type 2 inhibitor, as well as injection drugs such as insulin.

Through analyzing the data of 1172 T1D patients, a prospective study of European Diabetes identified that the incidence of neuropathy was associated with potential risk factors like the duration of diabetes, total cholesterol and LDL-C and triglycerides, body mass index, hypertension and smoking (Tesfaye et al., 2005). Studies demonstrated that in TID and T2D patients, DN might be prevented or delayed by the improvement in glycemic control (Ishibashi and Tavakoli, 2018; Mahelková et al., 2018). A clinical trial found that after application of insulin eye drops in 14 diabetic patients, the rate of corneal epithelial regeneration and the healing rate of epithelial defects increased (Bastion and Ling, 2013). In two animal studies, the authors reported that insulin eye drops could prevent corneal sub-basal nerve or sub-epithelial nerve loss in streptozotocin (STZ)-induced diabetic mice (T1D) (Chen et al., 2013; Yorek et al., 2014). In addition, Song et al. found that early insulin treatment was beneficial to normalise the circadian rhythm of the cornea in mice with T1D (Song et al., 2016). Moreover, in T1D rats, metformin treatment showed a protective function in hyperglycemia-induced ocular deteriorations like corneal epithelial defects and damaged Bowman’s membrane through ameliorating oxygenated free radicals in the endothelial cells by inhibiting protein kinase C, reducing mitochondrial respiratory chain pathways (Nahar et al., 2021). These data indicated that insulin might be used for the treatment of diabetic corneal defects. However, in T2D, the role of glycemic control in complications is controversial. One clinical trial found that in T2D subjects, treatment with exenatide plus pioglitazone or basic bolus insulin therapy for more than 12 months could significantly improve corneal nerve morphology, which was manifested by increased corneal nerve length and branch density, indicating the promotion of corneal nerve regeneration (Ponirakis et al., 2020). Nonetheless, another clinical trial demonstrated that treatment with exenatide combined with pioglitazone or insulin had a limited effect on nerve regeneration in T2D subjects with high insulin resistance (Ponirakis et al., 2021b). However, it should be noted that in non-diabetic cornea of rats, topical insulin had no effect on wound healing (Zagon et al., 2007). Also, insulin treatment may up-regulate insulin-like growth factor-1 (IGF-1), activate vascular endothelial growth factor production, increase the susceptibility to T2D and DR, and exacerbate retinopathy (Smith et al., 1999; Khan et al., 2020). Therefore, before insulin is considered as the treatment for diabetic corneal disorder, it is necessary to monitor the changes in retina.

Restoring insulin secretion is another means to treat diabetes and its complications. Pancreatic transplantation may be an effective way to restore normal glycemic (Tavakoli and Liong, 2012). A recent study demonstrated that in T1D patients, improvement in corneal nerve fiber regeneration was found after simultaneous transplantation of pancreas and kidney (Azmi et al., 2019). Nevertheless, suitable organs are limited, and surgery also brings huge risks and unpredictable postoperative complications. Hence, an alternative method, pancreatic islet cell transplantation has been considered to restore endogenous insulin production. Islet cell transplantation is a procedure to infuse purified islet cells from a deceased donor into the recipient’s liver. Therefore, it requires the use of immunosuppressive agents for life-long treatment (Senior et al., 2018). Overall, studies indicated that pancreatic islet cell transplantation may improve glycemic stability and may reduce the occurrence of diabetic complications (retinopathy and neuropathy) (Foster et al., 2018; Vantyghem et al., 2019). However, pancreatic islet cell transplantation can cause surgical complications such as infection, and the lifetime effectiveness is uncertain. An economic evaluation compared the cost of islet cell transplantation to insulin treatment and found that islet cell transplantation was not cost-effective (Wallner et al., 2016).

It was reported that worsening of DR occurred when diabetic patients changed from poor glycemic control to tight glycemic control (Bain et al., 2019), which emphasized the importance of monitoring blood glucose level. Recently, greater glucose variability was shown to be related to poor ocular nerve function, which was mentioned in *Clinical perspectives of corneal neuropathy in diabetes mellitus Section* (Issar et al., 2020). Therefore, more studies about treatments with less glucose variability in the prevention of neuropathy are needed.

### Pharmacological Intervention

#### Lipid-Lowering Drugs

Lipids include fatty acids, glycerolipids, sphingolipids and sterols (Fahy et al., 2005). In *Lipids Section*, we have concluded that dyslipidemia plays an essential role in DN. Moreover, a recent study revealed that T2D patients with metabolic syndrome manifested poorer peripheral nerve structure and function when compared with T2D patients alone (Issar et al., 2021), which again stressed the importance of lowering the level of harmful lipids.

Statins are the most used lipid-lowering drugs. According to published reports, statin treatment can improve neuropathic indicators by anti-inflammation in vincristine sulphate-induced DN in rats (Bhalla et al., 2015). A clinical trial revealed ​that simvastatin/ezetimibe and rosuvastatin could significantly reduce lipid peroxidation in patients with DN (Villegas-Rivera et al., 2015). However, other studies did not find significant association between statins and DN (Miao Wang et al., 2021). Fibrates are also used to lower triglyceride and increase high-density lipoprotein. Moreover, peroxisome proliferator-activated receptor (PPAR) played vital roles in modulating inflammation and metabolism. A drug in one of the fibrate classes, fenofibrate, is a PPARα agonist used to treat abnormal blood lipid levels. Knockout of PPARα in T1D mice and rats aggravated the reduction in corneal nerve fiber density and lowering in corneal sensitivity, which could be prevented by fenofibrate (Matlock et al., 2020).

Nevertheless, in T2D patients with/without DN, low levels of LDL-C could decrease nerve conduction velocity and amplitude and increase nerve lesions, despite being an effective treatment for dyslipidemia in T2D patients (Jende et al., 2019). A case report revealed that when PCSK9 inhibitor-alirocumab (Praluent) was used to treat patients with pre-diabetes and dyslipidemia, their level of LDL-C was reduced (Franco et al., 2019). The effects of triglyceride-lowering drugs on diabetic corneal neuropathy are largely unclear, and more well-designed experiments are warranted.

#### Supplements of Fatty Acids and Its Metabolites

Menhaden oil is a source of omega-3 fatty acids and is enriched in docosahexaenoic acid (DHA). It can be used as a dietary supplement for human (Shevalye et al., 2015). In both T1D and T2D rat models, the number of their corneal nerves was significantly reduced, indicating that the nerve fibers were excessively damaged. After treatment with a diet rich in menhaden oil, loss of corneal nerve was reversed, indicating that omega-3 fatty acids are a beneficial treatment (Coppey et al., 2020; Lewis et al., 2021). Another study found that in a T2D mouse model, the use of menhaden oil or resolvin D1 can reverse the attenuation in corneal innervation and motor and sensory nerve conduction velocity. In addition, through *in vitro* experiments, the author found that resolvin D1 stimulated neurite outgrowth in the dorsal root ganglion (Shevalye et al., 2015). Resolvin D1 is the metabolite of DHA and has anti-inflammatory and neuroprotective effects (Lee et al., 2015). DHA is involved in neuroprotection, synaptic function, and brain and retina development. DHA is the precursor of neuroprotectin D1 (NPD1), which could resist oxidative stress induced by cell injury (Serhan et al., 2015). One study reported that in injured rabbit cornea, treatment with NPD1 could attenuate inflammatory response, increase corneal sensitivity and stimulate neurite outgrowth ​(Cortina et al., 2013). In fact, corneas treated with DHA and pigment epithelium-derived factors exhibited increased NPD1 synthesis (Cortina et al., 2013).

Moreover, one article compared the effects of menhaden oil, resolvin D1 and salsalate on DN (Yorek et al., 2016). The authors found that in T1D mice, monotherapy could improve both motor and sensory nerve conduction velocities and increase the density of nerves in corneal epithelium, with no difference among the three drugs. Salsalate, a prodrug of salicylate, is used to treat pain and inflammation associated with rheumatic diseases in humans. Salsalate could reduce circulating triglycerides (Davidson et al., 2018), block vascular insulin resistance mediated by free fatty acid and reduce inflammation (Yorek et al., 2016) by reducing NF-κB and increasing nitric oxide to prevent ischemic nerve damage (McCarty, 2010). The authors found that the combined use of menhaden oil and salsalate could increase the level of plasma resolvin D1 when compared with that observed in monotherapy (Yorek et al., 2016). The authors put forward the hypothesis that under the same curative effect, the effective concentration of menhaden oil could be reduced to reach the highest standard of human dietary dose in order to increase its clinical feasibility.

Meanwhile, the combined use of enalapril, *a*-lipoic acid and menhaden oil in T2D rats could help the recovery of corneal sub-basal nerve fiber length and corneal sensitivity (Davidson et al., 2017). Alpha-lipoic acid is a free radical scavenger that can alleviate oxidative stress. Previous studies have shown that alpha-lipoic acid administration led to clinically significant improvement and prevention of neurological damage progression (Ziegler et al., 2011). Additionally, when combined with aldose reductase inhibitors, it could further enhance the improvement effect (Yorek et al., 2014). Enalapril is an angiotensin converting enzyme inhibitor that is widely used to treat hypertension and diabetic nephropathy. The authors hypothesized that the mechanism of this combination therapy might be a reduction of oxidative stress and inflammation and an increase omega-3 polyunsaturated fatty acids.

Therefore, the aforementioned fatty acids and its metabolites provide other options for potential new treatments for diseases involving corneal nerve injury.

#### Aldose Reductase Inhibitors

Aldose reductase is the rate-limiting enzyme in the polyol pathway, whose activation by hyperglycemia can cause corneal nerve injury. It is also a key promoter of inflammation and cytotoxic conditions. Therefore, in theory, aldose reductase inhibitor (ARI) can reduce corneal nerve injury and promote corneal epithelial regeneration *via* attenuating the activation of polyol pathway (Markoulli et al., 2018). Takamura et al. found that rats fed with galactose have corneal abnormalities like those in diabetic animals. Treatment with ARI (ranirestat) has been found to facilitate corneal wound healing in rats by reducing the expression of matrix metalloproteinase (MMP)-10 (Takamura et al., 2013).

However, the therapeutic effect of another ARI (CT-112) is controversial. One study reported that the topical application of CT-112 to 39 diabetic patients could reverse the abnormal morphology of corneal epithelial cells and reduce corneal sensitivity (Hosotani et al., 1995). Interestingly, a randomized double-masked clinical trial using CT-112 found that after 4 and 8 weeks of treatment, the healing of corneal epithelial wounds was accelerated, but no changes in corneal sensitivity were found (Nakahara et al., 2005).

Another double-masked clinical trial (Fujishima and Tsubota, 2002) found an effective improvement of corneal epithelial injury and recovery of corneal sensitivity with treatment of oral ARI (ONO-2235).

Hence, different ARIs may have different effects in diabetic patients, and more studies need to be conducted to determine the efficacy of individual drug. The effectiveness of combinational administration should be tested as well.

#### Trophic Factor Supplements

##### Insulin-like Growth Factor-1

IGF-1 is a peptide with multiple functions in central and peripheral nervous system. In both human and animal models of TID and T2D, the level of serum IGF-1 was significantly reduced (Aghanoori et al., 2019). The density of corneal sub-basal nerves in type 2 diabetic mice was reported to be reduced, and retrobulbar injections of IGF-I could increase nerve density and prevent corneal damage (Ueno et al., 2014). Using STZ-induced diabetic rats, one study revealed that IGF-1 enhanced mitochondrial function and drove axon growth through AMP-activated protein kinase, which prevented the death of distal fibers in DN (Aghanoori et al., 2019).

##### Nerve Growth Factor

NGF is a protein involved in the growth, differentiation, and survival of neurons. NGF, NT-3, NT-4, BDNF and GDNF are present in the cornea (You et al., 2000). Many studies have reported the association between NGF and ocular surface health. One study demonstrated (Lambiase et al., 2000) that NGF could facilitate the proliferation of corneal epithelial cells and accelerate epithelial wound healing in rabbits. During corneal wound healing, a variety of cytokines such as interleukin 1, were released to regulate the synthesis of NGF (Wilson et al., 1996). In mice with moderate to severe neurotrophic keratitis, topical NGF eye drops enhanced corneal epithelial healing, and improved corneal sensitivity and visual function (Bonini et al., 2000). Besides, damaged nerve repair and nutritional function abnormalities occurred in mice with vascular endothelial growth factor (VEGF)-B deficiency, which could be recovered with the administration of VEGF-B (Guaiquil et al., 2014). Indeed, SA treatment upregulated the expression of VEGF in cornea nerve after injury, thereby enhancing the repair ability of corneal nerve (Di et al., 2017). In another study, it was reported that exogenous VEGF-B could activate the PI-3K/Akt-GSK3β-mTOR signaling pathway. As a result, regeneration of diabetic corneal nerve fiber was promoted, with improved corneal sensitivity and a higher level of ​corneal pigment epithelial-derived factor (Li et al., 2017).

These findings indicated that neurotrophins were involved in the regulation of physiological and pathological processes on the ocular surface. It is therefore believed that NGF may become a potential therapeutic agent for ocular disorders. Numerous studies have investigated the use of recombinant human nerve growth factor (rhNGF). Yet, the role of rhNGF remained to be discussed. Several phase II or III clinical trials showed conflicting results on its early efficacy due to limited dosage testing (high dosage may cause severe side effects) and investigations in different populations (Apfel, 2002). Increasing the dose and at the same time reducing the adverse effects have become an urgent problem to be solved. One study reported that their laboratory could produce large amounts of rhNGF, whose pharmacological effects performed well without apparent side effects (Colangelo et al., 2005). This certainly provided supportive evidence for further clinical trials.

##### Neuropeptides

Neuropeptides co-exist with neurotransmitters in nerve cells, acting as both transmitters and trophic factors.

Substance P (SP) is a neuropeptide that acts as a neurotransmitter and neuromodulator and is mainly released by sensory nerve fibers. SP is present in the normal cornea in physiologically relevant concentrations (Nishida et al., 1996). The concentration of substance P in tears is thought to reflect the level of neuropeptides in ocular tissues (Suvas 2017). Compared with diabetic patients without DN, the SP concentration in tears of T1D patients with DN is significantly lower, and it was related to corneal nerve fiber density (Tummanapalli et al., 2019). Yet, there was no difference in substance P concentration in T2D patients with DN (Tummanapalli et al., 2020b). An article found that in T1D rats, reduction in hyporesponsiveness of C nerve fiber was found, which was related to the significantly decreased levels of substance P and calcitonin gene-related peptide (Rocha-Neto et al., 2019). One study revealed that in T1D mice, exogenous SP could accelerate skin wound healing, possibly because SP treatment induced an acute inflammatory response, which promoted cell proliferation and activated M2 macrophages (Leal et al., 2015). Thus, exogenous SP may be an effective treatment for neuropathy in diabetes. However, SP administration in rabbits had no significant effect on the healing of corneal epithelial wounds. But studies have found that topical co-treatment of SP and IGF-1 could facilitate the proliferation and migration of corneal epithelial cells in diabetic corneal neuropathy (Abdelkader et al., 2011).

C-peptide is a neuropeptide. During insulin biosynthesis, proinsulin is cleaved into insulin and C-peptide (Steiner et al., 1967). Due to the short biological half-life of C-peptide, Peg-C-peptide (a long-acting form of C-peptide) has been developed to increase patient compliance and facilitate translation into clinical applications. Jolivalt et al. found that T1D mice treated with C-peptide or Peg-C-peptide showed a significant increase in the corneal sub-basal nerve, but its specific mechanism was still unclear (Jolivalt et al., 2015).

Studies revealed that the synthesis of SP was regulated by NGF and exogenous NGF treatment in diabetic rats could restore nerve SP levels (Lindsay and Harmar, 1989). Meanwhile, C peptide could promote both NGF and SP levels (Kamiya et al., 2006). These results suggested that factors may interact with each other, whether the combination treatment can produce a synergistic effect deserves more research.

Another neuropeptide, GLP-1, has a role in inhibiting glucagon secretion and stimulating insulin secretion. It has been reported that GLP-1 receptor agonists had neuroprotective effects. In T1D mice, exendin-4 (a GLP-1 receptor agonist) could increase sensory and motor nerve conduction velocity and intraepidermal nerve fiber density as well as significantly promote neurite outgrowth from dorsal root ganglions (Himeno et al., 2011). However, in T2D mice, although exendin-4 improved motor nerve conduction velocity, it had no effect on sensory nerves (Kan et al., 2012). There is still a lack of research on the effect of GFP-1 on diabetic corneal nerves.

Vasoactive intestinal peptide (VIP) is an immunosuppressive neuropeptide that belongs to the glucagon superfamily. Recently, Zhang et al. found that the exogenous use of neuropeptides such as calcitonin gene-related peptide, SP and VIP could partially restore damaged corneal epithelial wounds and inhibit the overexpression of pro-inflammatory factors in T1D mice corneas. In addition, the authors further discovered that VIP up-regulated the expression of NGF, CNTF, anti-inflammatory cytokines and improved corneal epithelial wound healing and nerve regeneration through VIP/VIP type 1 receptor/Sonic Hedgehog pathway (Zhang et al., 2020b).

ARA 290 is a non-hematopoietic peptide designed based on the structure of erythropoietin. In one phase II clinical trial, treatment with ARA 290 in T2D patients markedly increased the mean corneal nerve fiber density when compared with the placebo group (Brines et al., 2015).

These studies showed that neurotrophic factors interact with neuropeptides. When the cornea is injured, their expression and function may form a positive feedback loop: NGF from the healed corneal epithelial cells and CNTF from the dendritic cells can induce regeneration of sensory nerve, which in turn cause the release of more neuropeptides to nourish and maintain the proliferation and migration of corneal epithelial cells and dendritic cells. DM disrupts this interaction, leading to corneal injury. Based on the published data, we hypothesized that combination treatment with both neurotrophic factors and neuropeptides might be a promising approach for diabetic corneal damage.

#### Antioxidants or Anti-inflammatory Drugs

##### Naltrexone

The endogenous opioid system affects the proliferation of many cells like neurons and glial cells. Opioid growth factor (OGF) has been detected in bovine and rabbit corneal epithelium and was found to have an inhibitory effect on the proliferation and growth of corneal epithelial cells (McLaughlin et al., 2010).

Naltrexone is a long-acting and potent opioid antagonist that can block the interaction of OGF and its receptor OGFr. In both *in vitro* and *in vivo* studies, treatment with naltrexone could significantly facilitate DNA synthesis, cell division, cell migration, corneal epithelial wound healing, and corneal epithelium proliferation and growth (Sassani et al., 2016). Interestingly, the combined topical use of naltrexone and insulin has no additive or synergistic effect on corneal epithelial regeneration in T1D rats (Klocek et al., 2009).

Although a lot of evidence demonstrated the effectiveness of naltrexone aqueous solution, there are very few reports on its ocular delivery system and therefore more research is needed for its use in clinical treatment of diabetic corneal damage.

##### Other Antioxidants

Antioxidants such as *ß*-carotene (Abdul-Hamid and Moustafa, 2014) and carnosine (Shevalye et al., 2015) have also been shown to have effects on diabetic corneal repair and can be used to combat the consequences of AGEs on the ocular surface. One recent study found that an antioxidant mitoquinone reduced the oxidative stress produced by mitochondria in nerves (Fink et al., 2020). Adding mitoquinone to the diet of diabetic rats accelerated nerve conduction velocity, increased corneal and intraepidermal nerve fiber density and restored corneal sensitivity.

Other drugs have also been investigated. 1,5-isoquinolinediol, an inhibitor of poly(ADP-ribose) polymerase, partially reversed the epithelial innervation loss, corneal sensitivity reduction and epithelial wound healing delay (Byun et al., 2015). Meanwhile, administration of KIOM-79 (a mixture of 80% ethanol extracts of parched Puerariae radix, gingered Magnoliae cortex, Glycyrrhizae radix and Euphorbiae radix) protected keratocyte from cell death, lessened oxidative stress in corneas *via* suppression of the AGEs/NF-κB signal pathway (Kim et al., 2011b).

#### Other Drugs

##### Vitamin B

One recent study reported that topical vitamin B12 and citicoline treatment could ameliorate the morphology and function of corneal nerves in diabetic patients, indicating a neuroprotective effect (Fogagnolo et al., 2020). Despite these promising results, more clinical trials need to be conducted to provide more evidence for the use of vitamin B in patients with diabetic corneal neuropathy.

##### Vitamin D

In recent years, several studies have focused on the relationship between vitamin D and DN. A clinical study involving nearly 10,000 subjects demonstrated that 50% of T2D patients had low serum vitamin D levels (Herrmann et al., 2015). Furthermore, a systematic review (involving 1,484 T2D patients) reported ​a marked association between vitamin D deficiency and the development of DN (Lv et al., 2015). Other studies revealed that the vitamin D level of painful DN patients was significantly lower than that of painless DN patients (Alam et al., 2021), and the corneal nerve fiber density decreased more significantly in patients with low vitamin D level (Almurdhi et al., 2017). The possible mechanism behind vitamin D’s effects is unclear. But previous animal studies have found that treating diabetic rats with a vitamin D analog promoted NGF production in their sciatic nerves (Riaz et al., 1999). Similarly, it has been found that Tacalcitol, an active vitamin D3, could up-regulate NGF level in human epidermal keratinocytes (Fukuoka et al., 2001). The involvement of growth factors is indicated, and further investigations are essential.

### Bariatric Surgery

Interestingly, two recent studies found an association between obesity and corneal nerve fibers. One study showed that in patients with severe obesity, early corneal nerve fiber and keratocyte density were attenuated (Iqbal et al., 2021). After bariatric surgery, corneal nerve regeneration was observed. Another study showed that in obese T2D patients, corneal nerve fiber length, corneal nerve fiber density and nerve branch density were significantly improved after bariatric surgery (Adam et al., 2021). These protection function may be related to an improvement in inflammation (Adam et al., 2021; Iqbal et al., 2021).

### Gene Therapy

Gene therapy has obvious potential in the treatment of corneal diseases because the cornea carries many advantages suitable for genetic research. One advantage is that the cornea has immune privilege, which means that it can tolerate antigens without evoking inflammatory immune responses. Another advantage is that the cornea can be cultured *in vitro* and maintained for several weeks, making it possible to optimize gene transfer to improve the effectiveness and safety of gene therapy. Nonetheless, due to the lack of information on the genetic causes of corneal diseases and the goals of gene therapy, clinical transformation is still slow. It has been reported that epigenetics may play a role in diabetic complications including corneal changes (Kowluru et al., 2015). DM may cause specific changes in gene expression levels and patterns, which may be corrected by gene therapy.

Viral gene therapy is an effective delivery tool for delivering specific genes to the cornea (Ljubimov and Saghizadeh, 2015). Nevertheless, traditional viruses may induce inflammation, immune responses, and uncontrolled virus integration. New recombinant adenovirus, adeno-associated virus and lentivirus are main types of viral vectors commonly used in cornea transfection, which will not evoke serious immune response and can transfect non-dividing cells. Studies have shown that both adenovirus and adeno-associated virus can deliver target genes into the human cornea successfully. However, compared with adeno-associated virus transduction, the transgene expression level with adenovirus transduction was much higher, which made it a good choice as a gene therapy vehicle (Liu et al., 2008).

In studies of gene therapy, the authors mentioned that in diabetic cornea, c-met is down-regulated and protease is up-regulated (Saghizadeh et al., 2014; Kramerov et al., 2016). Therefore, adenovirus gene therapy implemented with upregulation of c-met by gene overexpression as well as silencing of MMP-10 and cathepsin F by short hairpin RNA technology could improve the wound healing rate of diabetic cornea and restore the expression of some stem cell markers in the limbus. Combination therapy using these three adenoviruses had the best effect, and the EGFR-Akt and p38 pathways were found to be involved.

In addition, there are other gene therapy targets such as microRNAs to effectively normalize the pathologies in the diabetic cornea. Studies have found that miR-146a inhibitor (antagomir) promoted the wound healing of the cultured diabetic cornea by activating EGFR, and restored some stem cell expression markers (Winkler et al., 2014). Inspired by miRNA research, a study found that inhibition of miR-34c expression in T1D mice increased the expression of autophagy-related protein Atg4B in the trigeminal ganglion, and markedly promoted the growth of trigeminal sensory neurons and the regeneration of corneal nerve fibers (Hu et al., 2019). Interestingly, another study obtained the same experimental effect. It reported that subconjunctival injection of miR-181a antagomir in diabetic mice resulted in corneal nerve fiber regeneration and trigeminal sensory neuron growth through ATG5-mediated autophagy activation and BCL-2 mediated apoptosis inhibition (Hu et al., 2020). Although research related to microRNAs has made some progress, it should be noted that when compared with other targeted therapies that only affect specific genes or pathways, most miRNAs act on multiple targets and can simultaneously regulate many different genes in multiple pathways. This property may generate unpredictable side effects. Therefore, miRNAs treatment should be used cautiously and the delivery route and its biodistribution need to be repeatedly verified.

Nanotechnology also has potential applications in gene delivery. Viral vector has better transduction efficiency than nanocarriers like nanoparticles. Yet, many advantages, such as ease of synthesis and operation, low production cost, accommodation in large-size vectors, less inflammatory response, no risk for genomic insertion and mutation, made nanotechnology an attractive target (Han et al., 2012). Numerous studies on nanotechnology for corneal injury have emerged. It has been reported that dipotassium glycyrrhizinate-micelle formulation encapsulating active agents (Hou et al., 2021), nanoparticle-encapsulated Fidgetin-like 2 siRNA (Jessi Wang et al., 2021), and high-density lipoprotein nanoparticles with a gold nanoparticle core (Lavker et al., 2021) showed promising results in accelerating corneal re-epithelialization and anti-inflammation. In addition, poly (lactic-co-glycolic acid) nanoparticles could be a drug release system of Lingzhi to reduce oxidative damage in corneal epithelium cells (Tsai et al., 2021). In conclusion, nanotechnology is a potential therapeutic approach for gene transfer and drug delivery in diabetic corneal neuropathy.

In addition, using plasmid with PGC-1α promoter, using gene silencing and nanotechnology to knockdown CaMKKβ, the authors revealed that muscarinic toxin 7 augmented elevated mitochondrial function, neurite outgrowth in dorsal root ganglia neurons, and increased corneal nerve density through Ca^2+^/Calmodulin-Dependent Protein kinase *ß* (Saleh et al., 2020).

Overall, gene therapy may be a promising way to restore abnormalities of diabetic corneal but there is an urgent need for further studies in its clinical use.

## Summary

Diabetic corneal defects could be a biomarker for evaluating DN. Since studies (see *Clinical Perspectives of Corneal Neuropathy in Diabetes Mellitus Section*) showed that in diabetic patients, corneal defects commenced and showed a progressive degeneration as DM continued. Moreover, there was also reported a greater loss of corneal nerve fiber in DN patients when compared with diabetic subjects without DN. In addition, parameters of corneal nerve fibers worsened with increasing severity of DN. Therefore, it is essential to recognize and treat diabetic corneal neuropathy timely.

Firstly, since the complications of diabetes are caused by chronic hyperglycemia through a variety of pathological mechanisms, strict glycemic control is an important way to treat and reduce the progression of diabetic complications. Secondly, there have been many lipid-related clinical trials. Although the effects of triglyceride-lowering drugs such as statins and fibrates on diabetic corneal nerves are largely unknown, the fatty acids and their metabolites such as menhaden oil, resolvein D1, or their combined administration with free radical scavengers have provided alternative options for potential new therapies in diseases involving corneal nerve damage. Therefore, more elaborate and well-planned experimental and clinical studies are needed. Moreover, various ARI drugs have been shown to have different efficacy in diabetic patients, indicating the need for more clinical trials to determine what medication plan would yield better therapeutic effects. Growth factors such as IGF-1 and NGF have shown initial promising results in animal experiments. However, the reported serious side effects caused by high doses need to be addressed, since low doses have not achieved great efficacy in clinical trials. There were also many animal studies on neuropeptides such as SP and C-peptide, which reported that the combined therapy of neurotrophic factors and neuropeptides might be a promising therapeutic agent for ocular disorders. Meanwhile, vitamin B, vitamin D and naltrexone initially showed promising results. Yet, more clinical trials should be conducted to identify an effective ocular delivery system and provide evidence of their effects on diabetic corneal neuropathy. In addition, some progress has been made in microRNAs research. Due to the possibility of unpredictable side effects, miRNAs therapy should be used with caution, and their delivery methods and biodistribution need to be verified. Also, nanotechnology has become a promising therapeutic method for gene transfer and drug delivery. In general, gene therapy may be a potential method to restore diabetic corneal abnormalities, but again further clinical trials are needed.

## Conclusion

Diabetes mellitus has adverse effects on corneal nerve fibers, corneal sensitivity and corneal epithelial regeneration. Corneal nerve damage in diabetic patients usually precedes neuropathy in other tissues and is associated with the severity of DN, making it a potential early indicator of DN. Corneal confocal microscopy is an examination method that quantitatively evaluates the morphology of the corneal nerve, facilitating the use of corneal nerve plexus as an early biomarker to access DN. It is essential to increase the awareness of damage on diabetic ocular surface and take actions to timely detect diabetic corneal neuropathy and monitor its subclinical progress.

At the current stage, the prevention of corneal nerve damage and the promotion of corneal nerve regeneration have achieved positive preliminary results, but most of the published work involved animal models and some preliminary clinical studies. Further investigations on the mechanism of diabetic corneal neuropathy, potential biomarkers, and pathogen-oriented therapy need to be conducted.

## Abbreviations

AGEs, advanced glycation end products; ARI, aldose reductase inhibitor; BDNF, brain-derived neurotrophic factor; CCM, corneal confocal microscopy; CNTF, ciliary neurotrophic factor; DHA, docosahexaenoic acid; DM, diabetes mellitus; DN, diabetic neuropathy; DR, diabetic retinopathy; FFA, free fatty acid; GDNF, glial cell line-derived neurotrophic factor; GLP-1, glucagon-like peptide 1; IGF-1, insulin-like growth factor-1; LDL-C, low-density lipoprotein cholesterol; NADPH, nicotinamide adenine dinucleotide phosphate; NGF, nerve growth factor; NPD1, neuroprotectin D1; NT-3, neurotrophin-3; OGF, opioid growth factor; PKC, protein kinase C; PPAR, proliferator-activated receptor; rhNGF, recombinant human nerve growth factor; ROS, reactive oxygen species; SP, Substance P; STZ, streptozotocin; TID, type 1 diabetes; T2D, type 2 diabetes; VEGF, vascular endothelial growth factor; VIP, vasoactive intestinal peptide; WHO, World Health Organization.
